# Spin Energy Contributions of the Kinetic Energy Density in the Stabilization of the Metal–Ligand Interactions

**DOI:** 10.1021/acs.jpca.4c03334

**Published:** 2024-06-21

**Authors:** Pablo Carpio-Martínez, David I. Ramírez-Palma, Fernando Cortés-Guzmán

**Affiliations:** † Instituto de Química, Ciudad Universitaria, Ciudad de México, Universidad Nacional Autónoma de México, Ucú 04510, México; ‡ Centro Conjunto de Investigación en Química Sustentable UAEM-UNAM, Toluca-Atlacomulco, km. 14.5, C.P. 50200 Toluca, Estado de Mexicó, México; § Instituto de Química Unidad Mérida, Universidad Nacional Autónoma de México, Carretera Merida-Tetiz, Km. 4.5, Ucú 97357, México; ∥ Facultad de Química, Ciudad Universitaria, Ciudad de Mexicó, Universidad Nacional Autónoma de México, Ucú 04510, México

## Abstract

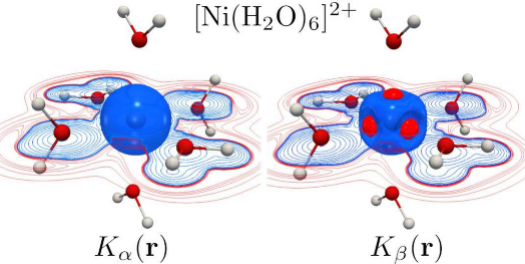

The kinetic energy (KE) density plays an essential role in the stabilization mechanism of covalent, polar covalent, and ionic bondings; however, its role in metal–ligand bindings remains unclear. In a recent work, the energetic contributions of the spin densities α and β were studied to explain the geometrical characteristics of a series of metal–ligand complexes. Notably, the KE density was found to modulate/stabilize the spin components of the intra-atomic nucleus–electron interactions within the metal in the complex. Here, we investigate the topographic properties of the spin components of the KE density for a family of high-spin hexa-aquo complexes ([M(H_2_O)_6_]^2+^) to shed light on the stabilization of the metal–ligand interaction. We compute the Lagrangian, *G*(**r**), and Hamiltonian, *K*(**r**), KE densities and analyze the evolution of its spin components in the formation of two metal–ligand coordination complexes. We study *K*_α/β_(**r**) along the metal–oxygen (M–O) internuclear axis as a function of the metal. Our results indicate that *K*(**r**) is a more distance-sensitive quantity compared to *G*(**r**) as it displays topographic features at larger M–O distances. Furthermore, *K*(**r**) allows one to identify the predominant interaction mechanism in the complexes.

## Introduction

In general, studying chemical interactions involves examining the valence region of the interacting species. Within the molecular orbital approach, the valence shell is usually defined by the set of orbitals with the highest principal quantum number energetically accessible to accept or donate electrons. However, within the context of real space analysis the valence shell demands an alternative definition since scalar fields are built upon every molecular orbital (not just the valence orbitals or a specific set of orbitals). In a previous work, our group analyzed the polarization of the valence shell of a metal in a complex in terms of the α and β components of the electron density.^1^ Furthermore, we recently studied the topology of the Hamiltonian kinetic energy density (KED) and its Laplacian to gain more insight into the role of kinetic energy in chemical interactions.^2^ We found that a covalent bond is characterized by a concentration of kinetic energy, potential energy, and electron densities along the internuclear axis. In this work, we study the local behavior of the spin components of the Lagrangian and Hamiltonian kinetic energy densities (within the valence shell of the metal center) in a series of coordination compounds. Moreover, we explain how classically allowed/forbidden motion of electrons might connect to the stabilization mechanism of the metal–ligand interaction.

### Kinetic Energy Density

There are two different points of view on the role of kinetic energy in forming chemical bonds. One view states that lowering the kinetic energy associated with electron delocalization is the key stabilization mechanism of covalent bonding.^3−15^ In contrast, the opposite view holds that a chemical bond is formed by a decrease in potential energy due to a concentration of electron density within the binding region.^16−20^ This latter has been recently supported by experimental and theoretical evidence comparing the free Be atom with that in a crystal environment.^21^ Classically, the local kinetic energy of a system can be defined without ambiguity; however, in quantum mechanics, there exist an infinite number of expressions that are integrated throughout the space and recover the total kinetic energy of the system. Most of these expressions are valid and mathematically justified; however, their conceptual usefulness is limited.^22−24^ Furthermore, based on the way the local kinetic energy is formulated, the spatial variation of the total energy is controlled by either the kinetic or potential energy.^25^ There exist several ways to generate a family of kinetic energy densities, but the most useful in chemistry comes from the additive multiple of the Laplacian of the electron density, i.e., the Laplacian family, defined as
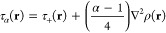
1where τ_+_(**r**) is the positive definite KED, which equals τ_1_(**r**) when α = 1, also denoted as *G*(**r**). When α = 0, one obtains the Schrodinger or Hamiltonian form, *K*(**r**). The expressions for these two KEDs are
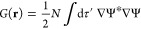
2
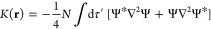
3The difference between these two functions emerges when one examines their local behavior. *G*(**r**) is finite and positive at all points in space and is generally a monotonic function in atoms. This convenient feature makes it easily tractable and is currently the most studied of the two functions; however, its use as a chemical descriptor is still limited due to its lack of structure.^26−28^ In contrast, *K*(**r**) can give rise to positive and negative values, contains information related to the charge distribution, and can be related to the magnitude of the momentum in coordinate space.^29^ Tachibana et al. studied *K*(**r**) along the course of a chemical reaction coordinate. Three atomic regions were identified based on a classical interpretation of the kinetic energy: *K*(**r** > 0) (*K*_*A*_), where the electron density is accumulated extensively and the motion of the electrons is guaranteed; *K*(**r** < 0) (*K*_*F*_), where the motion of an electron is classically forbidden; and *K*(**r** = 0) (*K*_*S*_), the boundary between the two previous regions, which gives the shape of the atoms involved in the reaction process. During a reaction, two *K*_*A*_ disjoint regions of neighboring atoms gradually polarized toward the internuclear region until they fuse, where the transition state can be identified with the coalescent point.^30−32^ Jacobsen argues that “a description of chemical bonding based on charge densities finds the essence of a chemical bond in answer to the question where electrons are while kinetic energy densities focus on where electrons stay.”^27^ Recently, we found that the Laplacian of the Hamiltonian KED exhibits a shell structure in atoms and that its outermost shell merges when a molecule is formed. We also found that a covalent bond is characterized by a concentration of kinetic energy, potential energy, and electron densities along the internuclear axis. In weak interactions, the external shells of the molecules merge into each other, resulting in an intermolecular surface comparable to that obtained by the analysis of noncovalent interactions (NCI).^2^

### Polarization of the Spin Valence Shell

Theories explaining the formation of metal–ligand interactions in coordination chemistry usually consider the disposition and relative energies of a set of empty and occupied metal d orbitals. One of them is crystal field theory^33^ which describes the breaking of d-orbital degeneracy due to the presence of the ligands. These approaches ignore the explicit role of the spin. However, it is possible to determine the role of the spin if we focus our attention on ∇^2^ρ(**r**) and the atomic graph, which is a topological object that summarizes the charge concentration and depletion in the valence shells of an atom in a molecule or metal within a complex.^1^ We found that an atomic graph is the result of catastrophe processes between the components α and β, where the local dominance of one spin shell over the other determines the final shape of the atomic graph and the disposition of the ligand in the coordination sphere. In this approach, it is possible to find the distance that determines the spin information communication between two chemical species. We also found that the separation of charge concentration in the valence shell of the metal, the shape of the atomic graph, is the result of the maximization of the nucleus–electron interactions in each spin shell to compensate for the emergence of the electron–electron repulsion concentrations.

Previous results show that during the metal–ligand approach of complexes [V(H_2_O)_6_]^2+^ and [Ni(H_2_O)_6_]^2+^, the water molecules donate 0.38*e* to vanadium, 0.07 α and 0.31 β, while nickel receives 0.51*e*, 0.16 α and 0.35 β.^1^ In the process, hydrogen atoms are the electron source, while oxygen atoms play a modulation role in filtering the densities α and β. The questions that arise here are: In what way do the spin components of the KE density behave? Does such behavior reveal a pivotal role in the metal–ligand interaction? To answer these questions, we analyze the local changes of *G*_α/β_(**r**) and *K*_α/β_(**r**) during the approach of water molecules to the metal and explore the behavior of such quantities in a family of hexa-aquo complexes.

### Electron Delocalization

The atomic electron populations, N(A), can be divided into two contributions based on the integration of the Fermi hole density: the electrons located within the atomic basin and those delocalized in the basins of other atoms. This electron partition is due to the motions of the same spin electrons correlated as expressed in eq 4, where *h*^α^ is the density of the Fermi hole for a α spin electron.^34^ The localized electron in an atomic region is determined by the fraction of the total possible Fermi correlation contained within the region Ω, *F*^α^(Ω,Ω), as the double integration of the Fermi hole density within Ω (eq 5). At the Hartree–Fock and DFT level, it is the sum of the squares of the overlap integrals of α spin orbitals *i* and *j* over Ω, *S*_*ij*_(Ω). Moreover, the extent of the delocalization of an electron between two atomic basins, *F*^α^(Ω,Ω*′*), is determined by the product of overlap integrals over both regions (eq 6).^34^
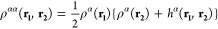
4
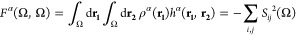
5
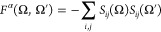
6

## Computational Details

In the case of the metal–ligand distance analysis for complexes [Ni(H_2_O)_6_]^2+^ and [V(H_2_O)_6_]^2+^ six water molecules were simultaneously located at different distances from the metal center (from 5.5 Å to the equilibrium distance of the complex with a step of 0.5 Å). These calculations were performed at the MP2^35^ theoretical level with the basis set def2-TZVPD^36^ to include the effects of electronic correlation. In the rest of the study cases, the hexa-aquo complexes [M(H_2_O)_6_]^2+^ at equilibrium metal–ligand distances, with M = Sc, Ti, V, Cr, Mn, Fe, Co, Ni, Cu, and Zn, were performed at the unrestricted DFT level using the functional PBE0^37^ with the same basis set. Even though pseudopotentials might be helpful in accounting for relativistic effects, we decided to keep a full-electron description of our systems to avoid any possible misrepresentation of the kinetic energy density as a consequence of the absence of core electrons.^38^ For this purpose, Gaussian 16 software^39^ was used, and all the coordination compounds were treated as high-spin systems. The NOAB, NOA, and NOB Gaussian keywords give the sets of natural spin orbitals. The 1D, 2D, and 3D grids were calculated for the spin components of the kinetic energy densities, *G*(**r**) and *K*(**r**), using the AIMALL package.^40^ The delocalization quantities were obtained using the same program. The contour diagrams were visualized with a script written in PvPython implemented in Paraview 5.9.^41^

## Results and Discussion

### Suitable Topographic Features for Metal–Ligand Interactions

Here, we show how the KE densities, *K*(**r**) and *G*(**r**), exhibit different topographic characteristics. Specifically, we demonstrate that *G*(**r**) does not reveal vital information between the spin components, as its behavior is similar to that of the electron density. In contrast, *K*(**r**) is endowed with (i) information about the distribution of charge, inherited by the Laplacian of the electron density, and (ii) the magnitude of the momentum throughout the coordinate space.^29^ The information contained in these two quantities is condensed in *K*(**r**) and is reflected in a highly structured landscape. We propose that this special feature might allow to pinpoint the role of the spin contributions. In addition, *K*(**r**) polarizes at long (5.5 Å) metal–ligand separation distances, making this KE suitable for detecting and characterizing coordination bond interactions. This sensitivity can be considered an advantage over the electron density since the effect on the polarization of the electron density and the transfer of spin information between the metal and the ligand is carried out at shorter distances, around 4 Å.^1^

We start by presenting the contour diagrams of *G*(**r**), *G*_α_(**r**), and *G*_β_(**r**) at different metal oxygen (M–O) separation distances for [Ni(H_2_O)_6_]^2+^ and [V(H_2_O)_6_]^2+^ (see Figures S1 and S2). In either case, at a distance of 5.50 Å, we observe an almost negligible polarization in the contours of the oxygen atoms. They appear circular and do not show any notable changes in the range of 5.50–4.0 Å and 5.50–3.50 Å for vanadium and nickel, respectively. Naturally, when the oxygen atoms approach sufficiently, their contours merge until they cover the whole molecule at the equilibrium distance. The first fusion of external oxygen contours for vanadium is observed in *G*_α_(**r**), between 4.0 and 3.50 Å, while for nickel, it is observed in *G*(**r**) between 3.5 and 3.00 Å. The latter may result from almost equal contributions of densities α and β. With respect to the metals, their total KE density contours show a subtle square-like polarization. This is mainly driven by *G*_α_(**r**) for the case of vanadium and *G*_β_(**r**) for the case of nickel.

In Figures S3 and S4, we present the contour diagrams of *K*(**r**), *K*_α_(**r**), and *K*_β_(**r**) at different separation distances of the M–O bond, where some differences can be observed with respect to their positive definite KE counterparts. First, the oxygen contours have a clear polarization starting at 5.5 Å, which is maintained throughout the metal–ligand approach. In terms of metals, the polarization is present at longer distances compared to *G*(**r**), starting at 5.5 Å for V and 4.5 Å for Ni. Second, changes in the vicinity of the metals appear to alter their signs (depending on the region) as the M–O distance reaches the equilibrium geometry. Third, the metal and oxygen contours merge at shorter distances than those of *G*(**r**), that is, 2.50–3.00 Å, for both metals. In general, one can attribute these changes to the nonclassical behavior of *K*(**r**), which contains information about the distribution of charge and the magnitude of the momentum at every point in space.^29^

**Figure 1:**
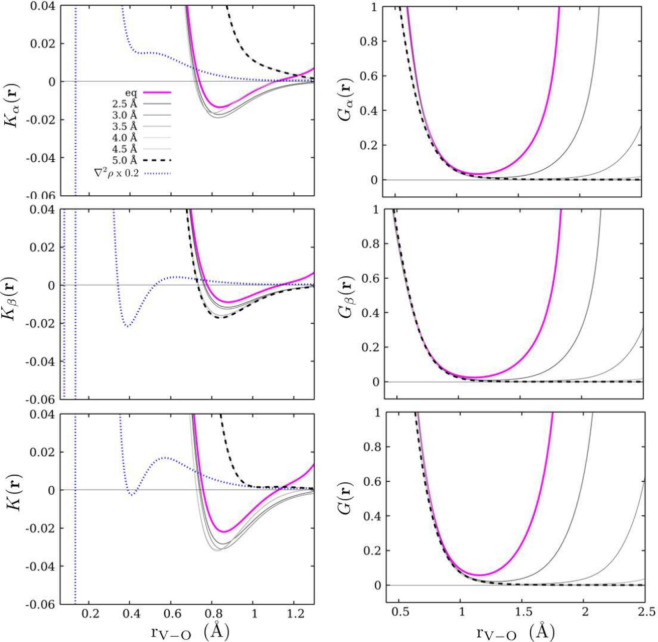
Profiles of the spin components of the Hamiltonian KED, *K*_α_(**r**) and *K*_β_(**r**), and Lagrangian KED, *G*_α_(**r**) and *G*_β_(**r**), for various V–O internuclear distances in the [V(H_2_O)_6_]^2+^ complex (top and middle panels, respectively). The bottom panels display the corresponding total kinetic energy densities, *K*(**r**) and *G*(**r**), for the same internuclear distances. ∇^2^ρ(**r**) is shown as a reference.

We calculated 1D profiles of *G*(**r**), *G*_α_(**r**), *G*_β_(**r**), *K*(**r**), *K*_α_(**r**), and *K*_β_(**r**) along the M–O internuclear axis at different separation distances. In all cases, we set the nuclear position of the metal as the origin while the position of oxygen was varied. First, we consider the [V(H_2_O)]^2+^ case shown in Figure 1. In the right panels, we display *G*(**r**), *G*_α_(**r**), and *G*_β_(**r**) where we observe dramatically low values and a monotonic increase in KE densities as one approaches the nuclear position of the metal. The *G*(**r**) minimum is located at approximately 1.2 Å in the equilibrium geometry. In the left panels, we present the profiles of *K*_α_(**r**), *K*_β_(**r**), and *K*(**r**) in which we observe several interesting characteristics. First, at 5.0 Å, the *K*_α_(**r**) profile shows a monotonic increase, going from oxygen to vanadium, while at the same distance, the *K*_β_(**r**) profile already exhibits a minimum. In equilibrium geometry, *K*_α_(**r**), *K*_β_(**r**), and *K*(**r**) have the same qualitative behavior, with negative values within the range of 0.7–1.1 Å, and minima located at almost the same distance (0.85 Å). For intermediate instances, the profiles are similar to those in equilibrium, with no substantial variation in the magnitude of the minima. These observations may provide an idea of the polarization sensitivity of the metal with respect to the spin components of *K*(**r**). The β component interacts at longer distances with vanadium before stabilizing the metal–ligand interaction.

**Figure 2:**
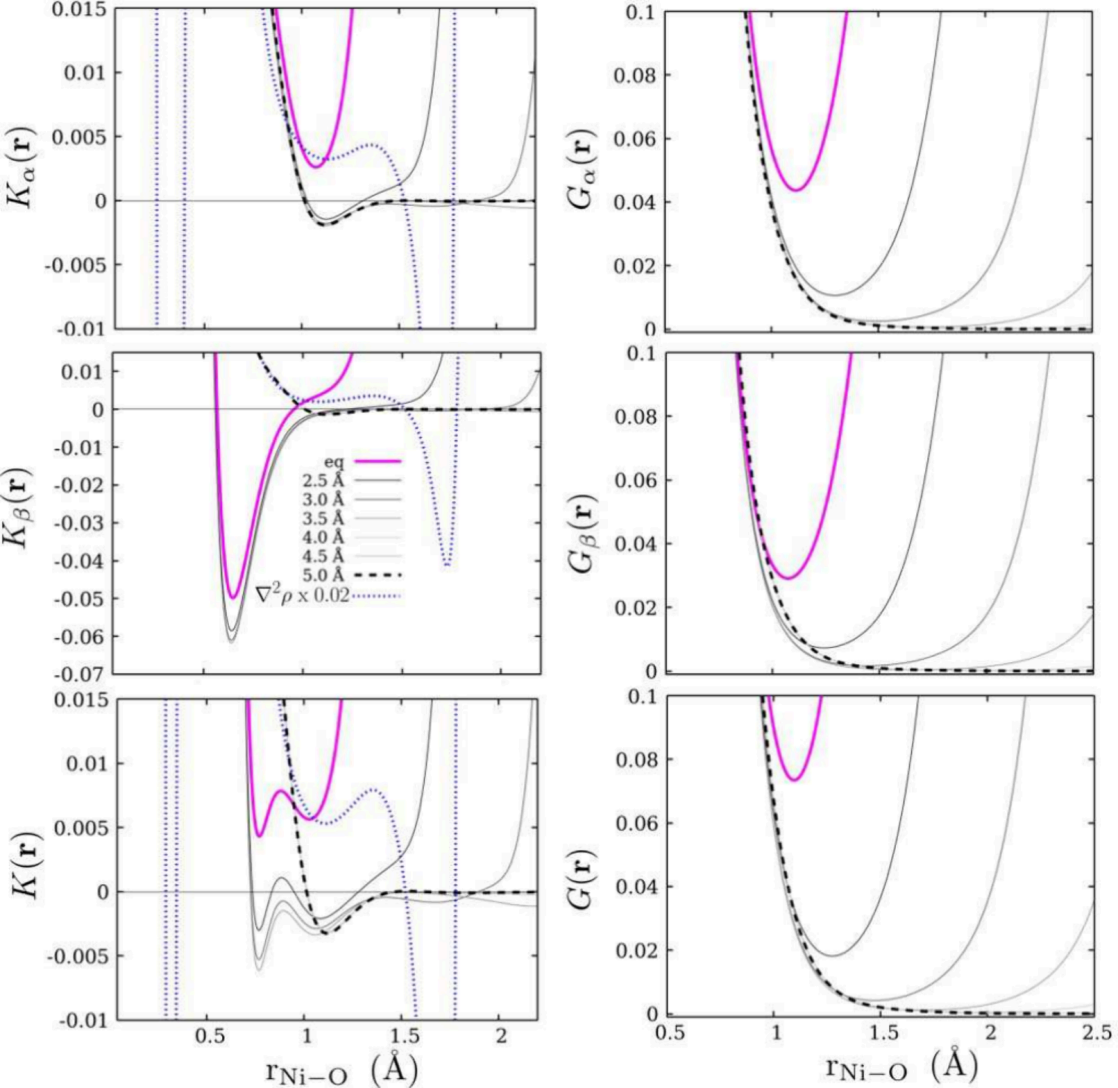
Profiles of the spin components of the Hamiltonian KED, *K*_α_(**r**) and *K*_β_(**r**), and Lagrangian KED, *G*_α_(**r**) and *G*_β_(**r**), for various Ni–O internuclear distances in the [Ni(H_2_O)_6_]^2+^ complex (top and middle panels, respectively). The bottom panels display the corresponding total kinetic energy densities, *K*(**r**) and *G*(**r**), for the same internuclear distances. ∇^2^ρ(**r**) is shown as a reference.

Next, we show the 1D profiles of *K*_α_(**r**) and *K*_β_(**r**) (see the left panels of Figure 2) for the [Ni(H_2_O)_6_]^2+^ complex. Here, we note that the density *K*_α_(**r**) has negative curvature at negative values when the M–O separation is 5.00 Å (contrary to what is observed in vanadium). In equilibrium geometry, the curvature is shifted to positive values with a minimum at ∼1.1 Å. With respect to β, the KE shows a drastic change (from 5.50 Å to equilibrium), going from a slowly monotonic curve to a negative curvature at ∼0.6 Å. Interestingly, the minimum in the latter is closer to the nuclear position of the metal compared to that in *K*_α_(**r**). Finally, *K*(**r**) shows two minima at positive values in the equilibrium geometry, which are a consequence of having the minima of the spin components at different distances.

In general, the behavior observed in *G*(**r**), *G*_α_(**r**), and *G*_β_(**r**) is comparable to that of ρ(**r**), and therefore its topological analysis may lead to equivalent characteristics. In contrast, the topography of *K*(**r**) enables a distinction between spin contributions to the metal–ligand interaction (even at long M–O separation distances), and thus more information can be extracted from its topographical analysis.

### Kinetic Energy and Metal–Ligand Stabilization Mechanisms

In the analysis below, we propose that the spin components of *K*(**r**) could reveal the stabilization mechanism in a coordination bond by studying their behavior along the M–O internuclear axis. Notably, we propose that classically allowed regions of KE translate into stabilization through localization of electrons along the M–O axis. On the other hand, classically forbidden values of KE reflect the delocalization of electrons toward nonbonding regions.

**Figure 3:**
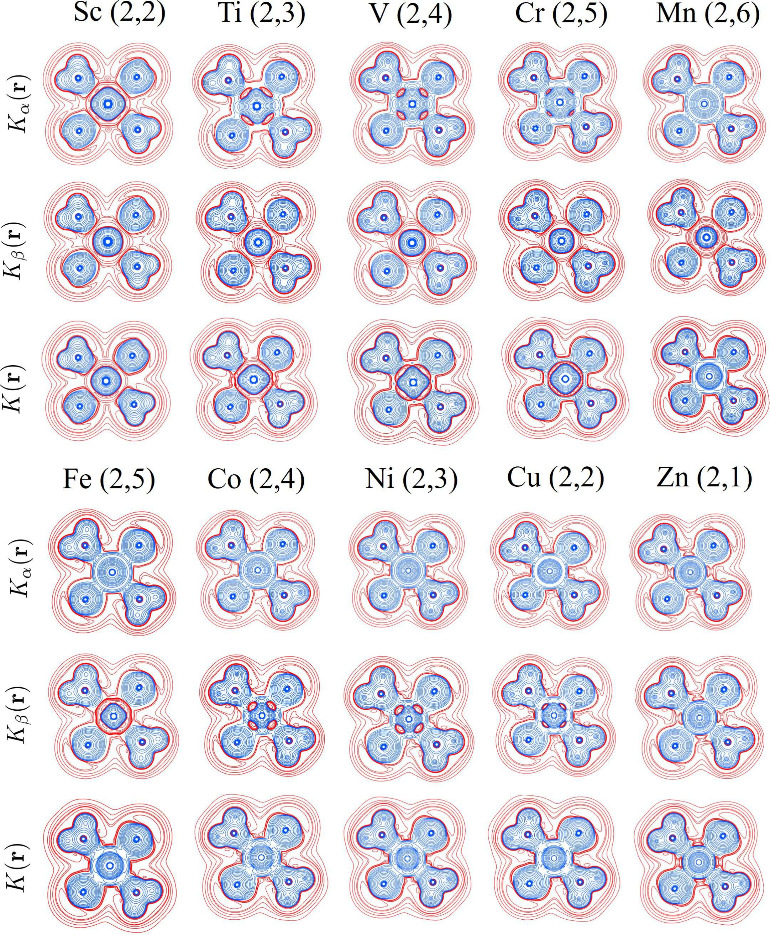
Contour diagrams of *K*_α_(**r**), *K*_β_(**r**), and *K*(**r**) in the equatorial plane for the [M(H_2_O)_6_]^2+^ complexes.

We focus on the topography of Hamiltonian KE densities in equilibrium geometry in a series of high-spin hexa-aquo complexes [M(H_2_O)_6_]^2+^, with M = Sc, Ti, V, Cr, Mn, Fe, Co, Ni, Cu, and Zn. We first analyze the contours of *K*(**r**) and its spin components in terms of the segmented regions defined by Tachibana.^31^ In Figure 3 we observe classically forbidden regions along the M–O internuclear axis, which gradually become positive as the atomic number of the metal increases, that is, as more electrons populate the orbitals of the metal center. Specifically, from Sc to Cr, classically forbidden regions are present in *K*(**r**), which come from their spin contributions. From Mn to Cu, *K*(**r**) is purely positive; however, the β component adopts negative values and gradually becomes positive as the atomic number increases. Finally, for the case of Zn, *K*(**r**) and its components are both positive. According to Ruedenberg’s picture of bond stabilization, the reduction of the KE (necessary for an energetically favorable interaction) is due to the ability of the electrons to delocalize between interacting fragments.^6,10,11^ This fact, in connection with Tachibana’s regions, might be used to deepen our understanding of the M–O interaction as follows. From Sc to Cr, the M–O interaction is mainly stabilized by the delocalization of electrons toward nonbonding regions because the electron motion between the metal center and oxygen atoms is highly forbidden. From Mn to Cu, more electrons populate the orbitals and positive contours emerge on the α component. This suggests a 2-fold stabilization. On the one hand, it is the delocalization caused by the classically forbidden motion of electrons in *K*_β_(**r**), and on the other hand, it is the localization caused by the classically allowed motion in *K*_α_(**r**).

**Figure 4:**
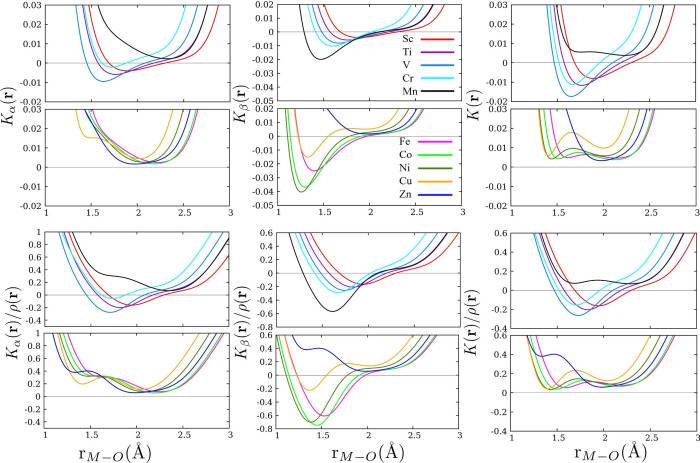
Top panels: *K*_α_(**r**), *K*_β_(**r**), and *K*(**r**) profiles along the M–O internuclear axis for the [M(H_2_O)_6_]^2+^ complexes. Bottom panels: profiles of *K*_α_(**r**)/ρ(**r**), *K*_β_(**r**)/ρ(**r**), and *K*(**r**)/ρ(**r**).

In Figure 4 (top panels), we present the profiles of *K*_α_(**r**), *K*_β_(**r**), and *K*(**r**) along the internuclear axis of the M–O bond. These plots show how the β component remains negative for all metals except Zn. In contrast, from Sc to Cr, the α component adopts negative values, and from Mn to Zn it spans over positive values only. Within the range of 1–3 Å, at least one minimum is observed in each KE density; nevertheless, the distance at which they appear is not the same from one spin component to the other. As a result, more structure is induced in the total density of KE *K*(**r**). For example, Ni has a minimum in *K*_α_(**r**) and a minimum in *K*_β_(**r**) at 2.1 and 1.2 Å, respectively. Therefore, the sum of both profiles is reflected in *K*(**r**) as it possesses two minima at around the same distances. In order to generalize the above analysis, we present the KE densities scaled to the value of the electron density at that point in space (see Figure 4, bottom panel). A direct consequence of dividing *K*(**r**) by ρ(**r**) is the appearance of new minimum values in the range of 1–2 Å in the α component. However, it is worth noting that this happens just for profiles that span positive values. Those that adopt negative KE are essentially unchanged; therefore, the ideas presented above are equally valid in this scenario.

**Figure 5:**
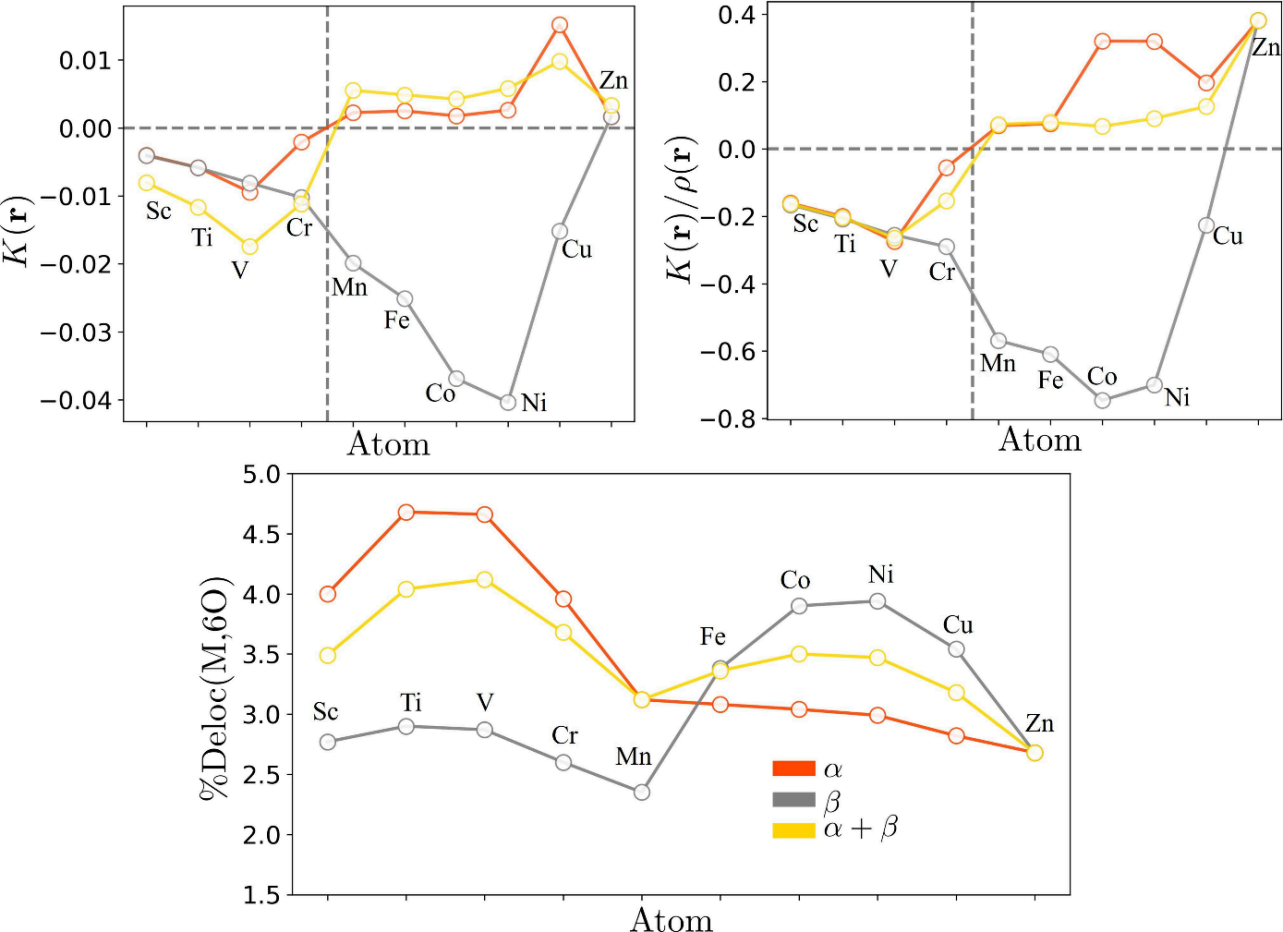
Left panel: minima of *K*_α_(**r**), *K*_β_(**r**), and *K*(**r**) along the M–O internuclear axis for the [M(H_2_O)_6_]^2+^ complexes. Right panel: minima of the quotients *K*_α_(**r**)/ρ(**r**), *K*_β_(**r**)/ρ(**r**), and *K*(**r**)/ρ(**r**). Bottom panel: percentage of electronic delocalization between the metal center and the 6 oxygen atoms of the water molecules, %Deloc(*M*,6*O*).

**Figure 6:**
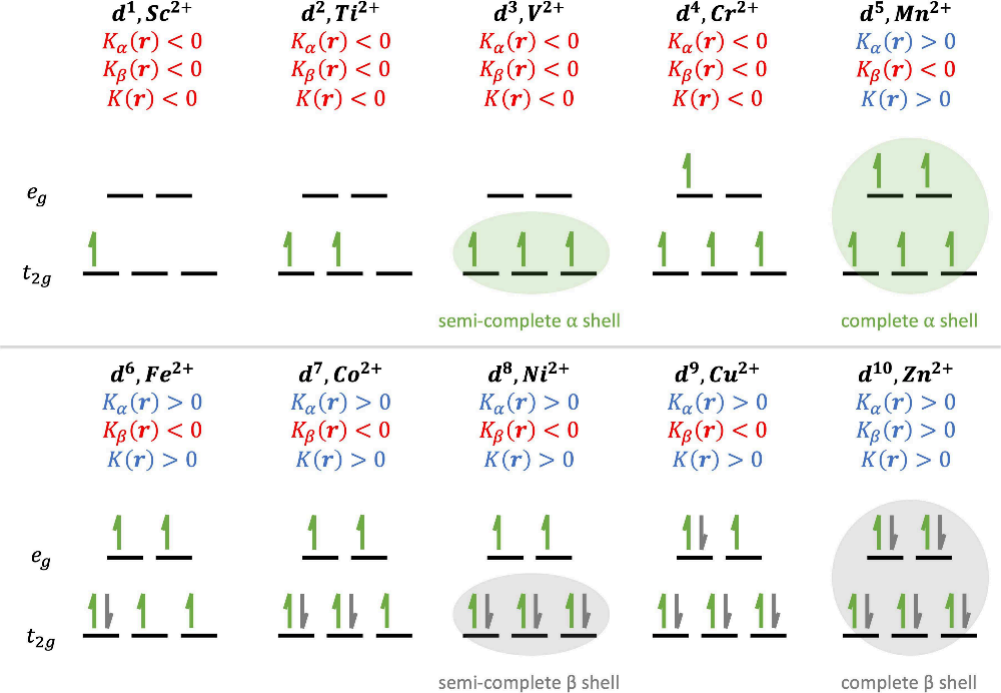
Electron occupation in d orbitals for octahedral complex splitting. The signs of the minima of *K*_α_(**r**), *K*_β_(**r**), and *K*(**r**) along the M–O internuclear axis for the [M(H_2_O)_6_]^2+^ complexes are indicated.

Finally, Figure 5 shows the minima values within the range of 1–3 Å for both *K*(**r**) and *K*(**r**)/ρ_β_(**r**). From Sc to Cr, negative minima are present in both spin components, with approximately the same magnitude. Starting from Mn, the minima exhibit opposite signs and different magnitudes, viz. positive for α and negative for β. For the latter, the minima tend to decrease as the atomic number increases, up to Ni in the case of *K*_β_(**r**) and up to Co in the case of *K*_β_(**r**)/ρ_β_(**r**). The minima of *K*(**r**) exhibit a behavior similar to that of the α component for both scenarios. Furthermore, some trends in Figures 3–5 can be related to the electronic configuration of the metal center but include spin information. For example, according to crystal field theory and the nature of d orbital splitting as a consequence of the electronic spin state of the metal center and the ligand,^42^ all complexes show a splitting of d orbitals for an octahedral geometry of 3 t_2g_ orbitals and 2 e_g_ orbitals, as shown in Figure 6. This implies that for a high-spin electronic configuration, there is a difference in property trends when the metal center presents a semicomplete or a fully occupied spin shell. This is in connection with the behavior observed in *K*_α_(**r**) with the increase in V to Cr, the change of sign in Mn, and the abrupt growth in Cu. On the other hand, in the case of *K*_β_(**r**), a decrease is observed up to the Ni atom, in the Cu atom there is an increase, and finally in Zn another change of sign occurs. However, global behavior does not completely follow this trend, and the change in sign in Mn seems to be the most important change. In this sense, a detailed analysis of the structure of *K*(**r**) and its spin components can provide more information on the stabilization of the metal–ligand interaction and complement other analysis of the metal–ligand bond. The behavior of these minima captures the trend of the metal–ligand interaction mechanism across the different metals.

Figure 5 also presents the delocalization between the metal and oxygen atoms as the percentage of the metal electron population (*N*(*M*)) delocalized within the oxygen atoms (*F*(*M*,*O*)), %Deloc = *F*(*M*,*O*)/*N*(*M*). From this figure, it is possible to observe that the delocalization maxima are presented with the *K*(**r**) minima. For V (semicomplete α shell) α delocalization is associated with the lowest *K*_α_(**r**) value. On the other hand, for Ni (semicomplete β shell) β delocalization is associated with the lowest *K*_β_(**r**) value. In general, positive values reflect the classically allowed motion of electrons and, therefore the localization of electrons, whereas negative values reflect the delocalization toward nonbinding regions.

## Conclusions

In a coordination bond, the polarization of the valence shell at the metal center is fundamental. In this work, we analyze the changes in the densities of kinetic energy, *G*(**r**) and *K*(**r**), in the region of atomic valence. The topographic analysis of *G*(**r**) and its spin components does not provide sufficient information to describe the metal–ligand interaction. On the other hand, *K*(**r**) is endowed with more topographical features that allow one to pinpoint the contribution of its spin components. Depending on the metal center, the topography of *K*(**r**) reveals how metal–ligand interactions (along the internuclear axis) are governed by regions in which electronic motion is allowed or forbidden. For Sc to Cr, the motion of electrons is not classically allowed; thus, the M–O interaction is mainly governed by delocalization toward nonbonding regions. From Mn to Cu, the α component stabilizes the M–O interaction by the existence of positive KE regions where the motion of electrons is guaranteed, while the β component stabilizes through delocalization. Finally, localization is the main source of stabilization in the Zn complex. Our findings not only explain the metal–ligand interactions in terms of the spin components of the KED but also may offer a conceptual use of *K*(**r**) in the description of chemical bonds.

## Supporting Information Available

Contour diagrams of *G*(**r**), *G*_α_(**r**), *G*_β_(**r**), *K*(**r**), *K*_α_(**r**), and *K*_β_(**r**) at different metal–oxygen (M–O) separation distances for [Ni(H_2_O)_6_]^2+^ and [V(H_2_O)_6_]^2+^ (PDF)
