# Supplementary material for: Spin Energy Contributions of the Kinetic Energy Density in the Stabilization of the Metal–Ligand Interactions
Source: J Phys Chem A. 2024 Jun 21;128(26):5158–65. doi: 10.1021/acs.jpca.4c03334 (PMC11229002; doi:10.1021/acs.jpca.4c03334)
Supplement: Supplementary file 1 — jp4c03334_si_001.pdf [file jp4c03334_si_001.pdf]

Spin Energy Contributions of the Kinetic Energy Density in the Stabilization of the Metal-Ligand Interactions

[†]Pablo Carpio-Martínez, [†]David I. Ramírez-Palma, [‡]Fernando Cortés-Guzmán

[†]Universidad Nacional Autónoma de México, Instituto de Química, Ciudad Universitaria, Ciudad de México, 04510, México

[‡]Universidad Nacional Autónoma de México, Facultad de Química, Ciudad Universitaria, Ciudad de México, 04510, México

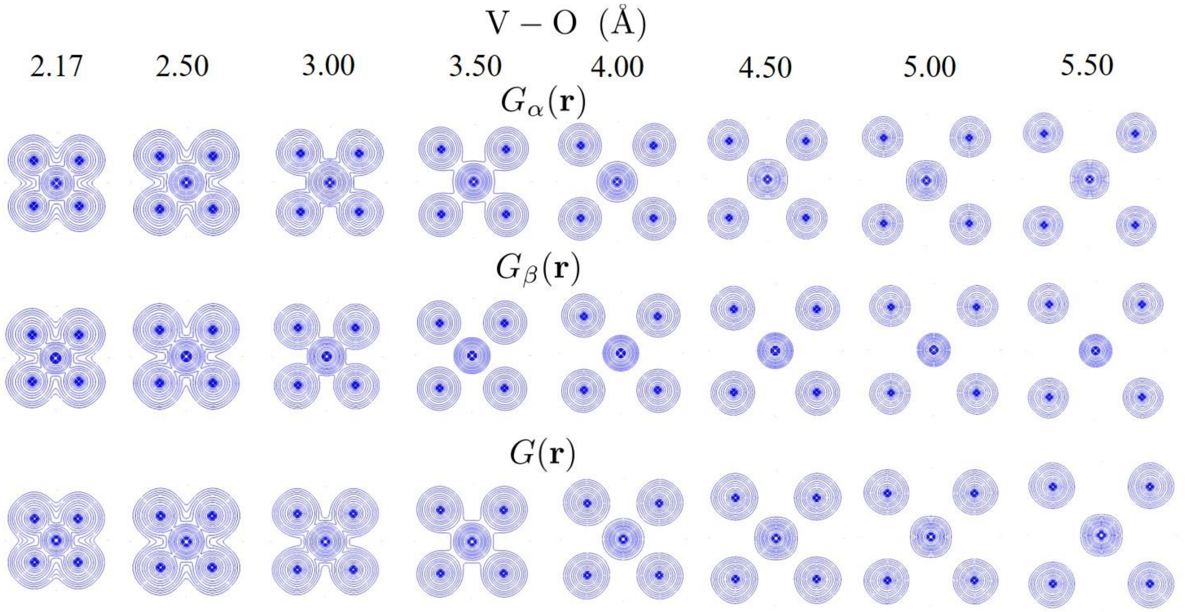

Figure S1. Evolution of the G(**r**) contours during the formation of the $[V(H_2O)_6]^{2+}$ complex.

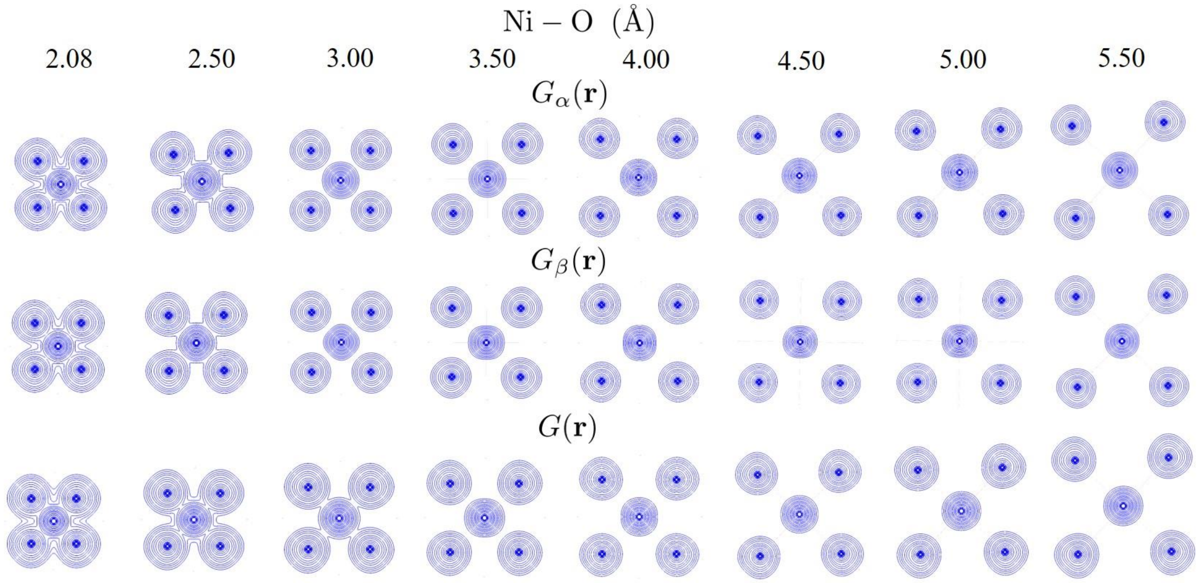

Figure S2. Evolution of the G(**r**) contours during the formation of the $[Ni(H_2O)_6]^{2+}$ complex.

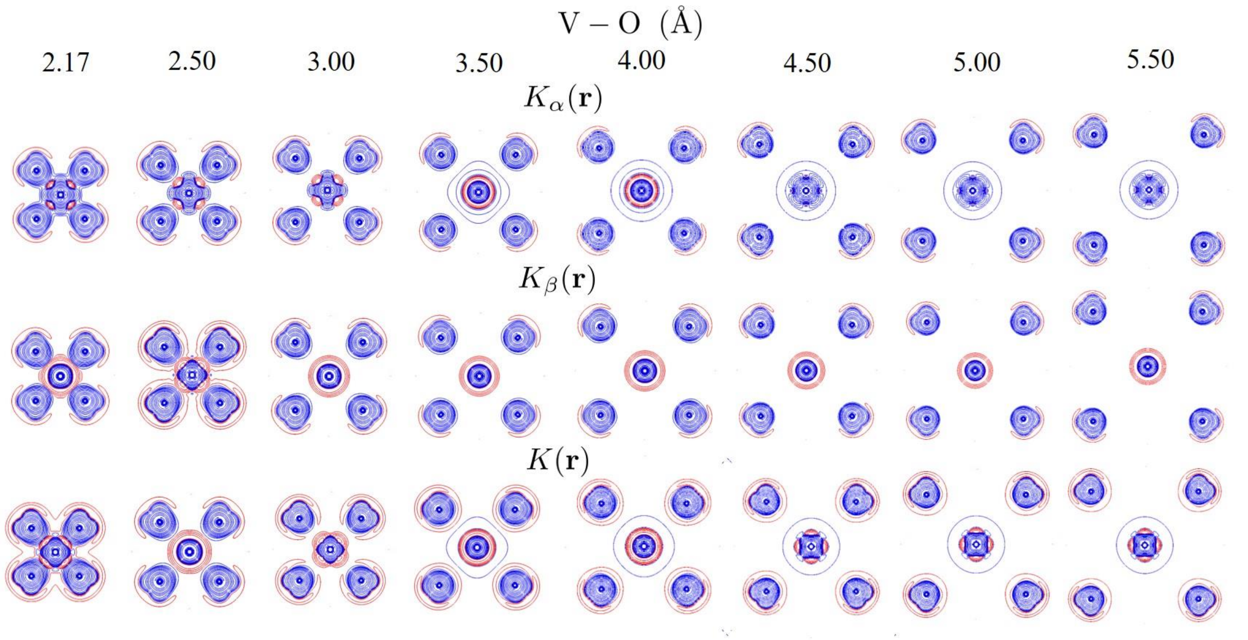

Figure S3. Evolution of the K(**r**) contours during the formation of the $[V(H_2O)_6]^{2+}$ complex. Positive values in blue and negative values in red.

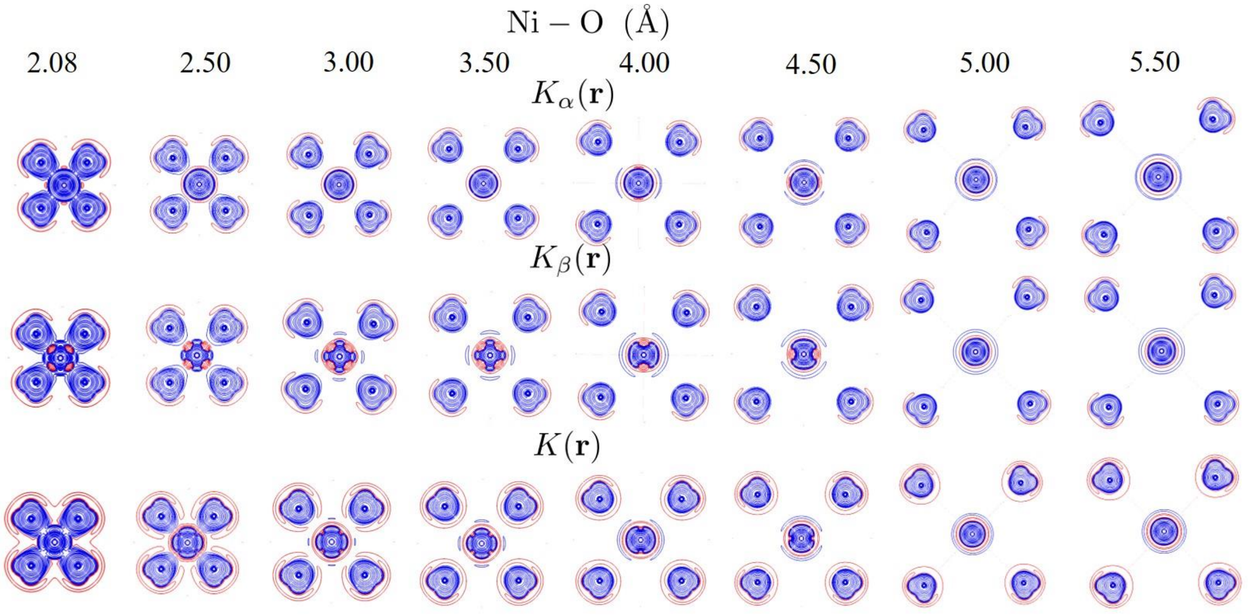

Figure S4. Evolution of the K(**r**) contours during the formation of the $[Ni(H_2O)_6]^{2+}$ complex Positive values in blue and negative values in red.
